# Genetic influences on externalizing psychopathology overlap with cognitive functioning and show developmental variation

**DOI:** 10.1192/j.eurpsy.2021.21

**Published:** 2021-03-31

**Authors:** Josephine Mollon, Emma E. M. Knowles, Samuel R. Mathias, Amanda Rodrigue, Tyler M. Moore, Monica E. Calkins, Ruben C. Gur, Juan Manuel Peralta, Daniel J. Weiner, Elise B. Robinson, Raquel E. Gur, John Blangero, Laura Almasy, David C. Glahn

**Affiliations:** 1 Department of Psychiatry, Boston Children’s Hospital, Harvard Medical School, Boston, Massachusetts, USA; 2 Brain Behavior Laboratory, Department of Psychiatry, Perelman School of Medicine, Penn-CHOP Lifespan Brain Institute, University of Pennsylvania, Philadelphia, Pennsylvania, USA; 3 South Texas Diabetes and Obesity Institute, School of Medicine, University of Texas of the Rio Grande Valley, Brownsville, Texas, USA; 4 Analytic and Translational Genetics Unit, Department of Medicine, Massachusetts General Hospital and Harvard Medical School, Boston, Massachusetts, USA; 5 Stanley Center for Psychiatric Research, Broad Institute of MIT and Harvard, Cambridge, Massachusetts, USA; 6 Program in Medical and Population Genetics, Broad Institute of MIT and Harvard, Cambridge, Massachusetts, USA; 7 Department of Genetics, Perelman School of Medicine, Penn-CHOP Lifespan Brain Institute, University of Pennsylvania, Philadelphia, Pennsylvania, USA; 8 Olin Neuropsychiatry Research Center, Institute of Living, Hartford, Connecticut, USA

**Keywords:** Cognition, development, externalizing, Gene × Age, heritability, pleiotropy, psychopathology

## Abstract

**Background:**

Questions remain regarding whether genetic influences on early life psychopathology overlap with cognition and show developmental variation.

**Methods:**

Using data from 9,421 individuals aged 8–21 from the Philadelphia Neurodevelopmental Cohort, factors of psychopathology were generated using a bifactor model of item-level data from a psychiatric interview. Five orthogonal factors were generated: anxious-misery (mood and anxiety), externalizing (attention deficit hyperactivity and conduct disorder), fear (phobias), psychosis-spectrum, and a general factor. Genetic analyses were conducted on a subsample of 4,662 individuals of European American ancestry. A genetic relatedness matrix was used to estimate heritability of these factors, and genetic correlations with executive function, episodic memory, complex reasoning, social cognition, motor speed, and general cognitive ability. Gene × Age analyses determined whether genetic influences on these factors show developmental variation.

**Results:**

Externalizing was heritable (*h^2^* = 0.46, *p* = 1 × 10^−6^), but not anxious-misery (*h^2^* = 0.09, *p* = 0.183), fear (*h^2^* = 0.04, *p* = 0.337), psychosis-spectrum (*h^2^* = 0.00, *p* = 0.494), or general psychopathology (*h^2^* = 0.21, *p* = 0.040). Externalizing showed genetic overlap with face memory (*ρ_g_* = −0.412, *p* = 0.004), verbal reasoning (*ρ_g_* = −0.485, *p* = 0.001), spatial reasoning (*ρ_g_* = −0.426, *p* = 0.010), motor speed (*ρ_g_* = 0.659, *p* = 1x10^−4^), verbal knowledge (*ρ_g_* = −0.314, *p* = 0.002), and general cognitive ability (*g)*(*ρ_g_* = −0.394, *p* = 0.002). Gene × Age analyses revealed decreasing genetic variance (*γ_g_* = −0.146, *p* = 0.004) and increasing environmental variance (*γ_e_* = 0.059, *p* = 0.009) on externalizing.

**Conclusions:**

Cognitive impairment may be a useful endophenotype of externalizing psychopathology and, therefore, help elucidate its pathophysiological underpinnings. Decreasing genetic variance suggests that gene discovery efforts may be more fruitful in children than adolescents or young adults.

## Introduction

Psychiatric symptoms in early life are associated with poor cognition [1]. For example, psychotic symptoms in childhood and adolescence are associated with cognitive impairment [2,3]. There is also evidence for IQ deficits in children with conduct problems [4], vocabulary deficits in children with aggression [5], visuospatial deficits in children with hyperactivity [6], and social deficits in adolescents with externalizing problems [7]. Small, generalized deficits have also been reported in children with symptoms of anxiety and depression [8].

Early life psychopathology is also underpinned by genes with evidence of substantial genetic effects on childhood and adolescent psychopathology [9,10], as well as specific psychotic [11,12], externalizing [13–15], internalizing [16], and anxiety/depression symptoms [17–21]. Since cognition in early life is also influenced by genes [22,23], recent studies have examined whether there is a genetic component to cognitive correlates of early life psychopathology. Evidence from twin and family studies shows genetic overlap between psychopathology and cognitive ability [24], psychopathology and executive functions [25], attention deficit hyperactivity disorder (ADHD) and IQ [26], ADHD and executive functions [27,28], and inattention and attention regulation [29]. GWAS evidence shows genetic overlap between ADHD and intelligence [30-32], academic underperformance [31,33,34], and executive functions [35]. However, a comprehensive examination of genetic underpinnings of cognitive impairment in early life psychopathology, that is, comprising multiple dimensions of psychopathology and cognition, is lacking.

Gaps in knowledge also exist regarding whether genetic influences on early life psychopathology show developmental variation. A recent review reported static and dynamic effects on internalizing and externalizing symptoms between infancy and early adulthood [13], with certain genetic effects remaining influential throughout development, and novel genetic factors also emerging. However, previous studies focused on developmental periods rather than examining age continuously. Another way to test for developmental variation in genetic influences is Gene × Age (G × A) interactions. G × A interactions can be tested using a cross-sectional design that models differences in psychopathology as a function of relatedness and similarity in age between individuals [36–38]. Thus, G × A analysis tests for fluctuations in action of genetic factors, as well as variation in genetic factors at different ages [23].

We used data from the Philadelphia Neurodevelopmental Cohort (PNC) [1], a large population-based sample aged 8–21, to derive factors of psychopathology using a bifactor model of item-level data from a psychiatric interview [39,40]. In bifactor models, items load on two factors simultaneously: (a) a general factor that accounts for commonality of all items (here general psychopathology) and (b) specific factors that account for unique influence of specific domains over and above the general factor (specific dimensions of psychopathology) [41,42]. Thus, bifactor models estimate the contribution of items to the general factor after controlling for specific factors, and vice versa [39]. Therefore, the utility of bifactor models lies in their ability to parse out this shared variance between general and specific factors, such that there is no contamination between factors, meaning that the general factor cannot account for findings in the specific factors, or vice versa. In other words, bifactor models allow examination of the unique contribution of the general and specific factors to prediction of external factors, or of the unique contribution of external factors (here genetic factors) to the general and specific factors [43,44]. Importantly, bifactor models accommodate orthogonal factor scores despite correlated latent factors [39,43]. We then used a genetic relatedness matrix to establish whether these psychopathology factors (a) were heritable, (b) showed genetic overlap with cognition, and (b) showed G × A interactions. In line with previous evidence, we hypothesized that psychopathology factors, would be (a) heritable, (b) show negative genetic correlations with cognitive functioning, and (c) be influenced by developmentally dynamic genetic factors, that is, show G × A interactions.

## Methods

### Participants

PNC is a population-based sample from the greater Philadelphia area, comprising 9,421 individuals aged 8–21. The study has been described in detail [1]. Briefly, between 2006 and 2012, 50,293 adults were recruited by the Center for Applied Genomics at Children’s Hospital of Philadelphia and provided access to Electronic Medical Records (EMRs). EMRs were screened for eligibility for PNC participation, yielding 19,161 individuals, released to the recruitment team in weekly waves between 2009 and 2011. Potential probands (ages 18–21) or caregivers/legal guardians (ages 8–17) were sent letters introducing the study, and then contacted by phone to explain the study, verify eligibility, and schedule appointments. Participants provided written consent for genomic studies upon providing blood samples during the clinical visit. Inclusion criteria were: (a) ability to provide signed informed consent (parental consent for participants <18), (b) English language proficiency, and (c) physical and cognitive ability to participate in cognitive testing. Data are in dbGaP (https://www.ncbi.nlm.nih.gov/projects/gap/cgi-bin/study.cgi?study_id=phs000607.v3.p2).

Genetic analyses were limited to participants who identified as white non-Hispanic (European American), leaving 4,662 subjects with genetic, cognitive, and psychiatric data. Mean age was 13.8 (standard deviation [SD] = 3.6), 50.3% were male (*n* = 2,346).

### Cognitive assessment

Participants completed the Penn computerized neurocognitive battery [45,46], which consists of 14 tests that capture functioning in five domains: (a) executive function (abstraction and mental flexibility, attention, working memory), (b) episodic memory (verbal, facial, spatial), (c) complex cognition (verbal reasoning, nonverbal reasoning, spatial processing), (d) social cognition (emotion identification, emotion differentiation, age differentiation), and (e) speed (motor, sensorimotor). Accuracy and reaction times are recorded for each test. All tests show moderate to very high reliability [47]. The battery also included the reading subtest of the Wide Range Achievement Test (WRAT), a measure of general verbal knowledge. As in our prior work [23], we derived a general composite score (*g*) as the first component of principal component analysis (PCA) of accuracy scores. We also derived a general composite score for speed (*gs*) as the first component of PCA of reaction times. To minimize the impact of missing data, multivariate imputation by chained equation (MICE) [48] was used to impute missing values using the *mice* package in R [49]. Imputation was based on age, sex and available cognitive data (participants missing <50% cognitive data) [23]. Subsequent analyses were conducted on imputed data.

### Psychopathology assessment

Psychiatric symptoms were ascertained using a computerized, structured interview (GOASSESS) [1,39,50], a modified version of the Kiddie-Schedule for Affective Disorders and Schizophrenia [51]. GOASSESS was administered to caregivers/legal guardians (ages 8–10), participants and caregivers/legal guardians (ages 11–17), and participants (ages 18–21). Bachelor- and Master-level assessors underwent a 25-h training protocol comprising didactic sessions, assigned readings, and supervised pairwise practice. Assessors were certified through standardized procedures requiring observation by a certified clinical observer who rated proficiency on a 60-item checklist of interview procedures. Responses coded by the assessor were required to correspond to responses coded by a certified clinical observer. Assessors underwent repeat observation until meeting passing criteria [1].

### Factor analysis to create factors of psychopathology

**Figure 1.**
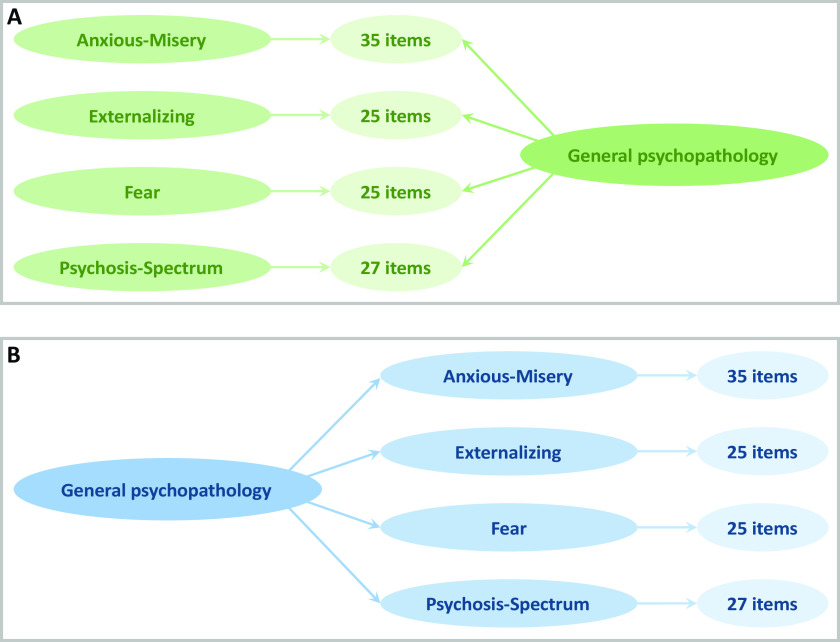
Schematic of (a) bifactor and (b) hierarchical models of 112 items from the GOASSESS structured interview.

We applied a confirmatory bifactor model [43] in Mplus [52] to 112 items from the GOASSESS [1,39,40](Figure 1a) using mean- and variance-adjusted weighted least squared estimator. Five orthogonal factors of psychopathology were generated for 9,421 individuals with GOASSESS data: (a) anxious-misery (mood and anxiety), (b) externalizing (ADHD and conduct disorder), (c) fear (phobias), (d) psychosis-spectrum, and (e) a general factor of overall psychopathology. Since bifactor models estimate the contribution of items to an overall dimension (general psychopathology) after controlling for specific factors, and vice versa, all factors (both general and specific) are orthogonal. Thus, bifactor models parse out the shared variance between general and specific factors, such that there is no contamination between factors. Therefore, bifactor models allow examination of the unique contribution of external factors (here genetic factors) to the general and specific factors [43,44]. Table S1 shows factor loadings, Table S2 shows correlations between factors (and with cognition), Figure S1 shows test information plots.

### Genotyping

Samples were genotyped on one of four Illumina arrays: HumanHap550, HumanHap610, OmniExpress, or Human1M. Genotyped data were imputed at the Broad Institute [53] using IMPUTE2 and reference haplotypes in Phase I of the 1000 genomes data (June 2011 release) that included ~37,138,905 variants from 1,094 individuals from Africa, Asia, Europe, and the United States. Imputed genotype data were used in subsequent analyses.

### Estimation of genetic relatedness matrix

Empirical relatedness quantifies the proportion of alleles that are identical by descent between individuals. Empirical relatedness was calculated for all pairs of individuals using genotype data [23]. Briefly, 50k common autosomal single nucleotide proteins (SNPs) in approximate linkage equilibrium were selected from all available SNP variants after linkage disequilibrium (LD) pruning (*r*^2^ > 0.1) using PLINK [54]. Relatedness was estimated from these SNPs using IBDLD [55](up to 50 SNPs within a 2-cm span). The matrix was inspected to ensure correct properties (trace equal to number of genotyped subjects, symmetry, positive semi-definiteness, range of diagonal, and off-diagonal elements). Distribution of estimated relatedness values has been presented previously [23]. Empirical genetic relatedness matrices are advantageous because quantitative genetic analyses (described below) that are traditionally applied to family data using genetic relatedness matrices calculated from pedigree information can, in turn, be applied to cohorts of related and/or unrelated individuals (such as the PNC) using empirical genetic relatedness instead.

### Statistical and quantitative genetic analyses

R [56] was used to generate descriptive statistics and graphics. Genetic analyses were conducted in Sequential Oligogenetic Linkage Analysis Routines (SOLAR) [57] on 4,662 participants who identified as white non-Hispanic (European American), regardless of relatedness. While analyses in SOLAR performed on family data are robust to population stratification, the PNC sample comprises both related and unrelated individuals. Therefore, we only included individuals of European American ancestry (the most common ancestry group in the PNC sample) in our analyses and excluded individuals of non-European ancestry. Relatedly, since rare variants that may explain a substantial proportion of phenotypic variance are not well captured by common SNPs, using related individuals is more powerful than using unrelated individuals when estimating heritability, but the combination of related and unrelated individuals, as in the PNC, is optimal. Moreover, related individuals (even distantly) are critical for detecting G × A interactions (described below). When using only unrelated individuals it is not possible to detect changes in genetic correlation over time since related pairs serve as a pseudo-longitudinal design where the same polygenotypes are observed at different ages.

### Univariate and bivariate polygenic models

SOLAR implements linear mixed-effects models, which decompose the overall variance of a quantitative trait [58,59]. Traditionally, these analyses are performed on family data using matrices calculated from pedigree information, but can be applied to cohorts of related and unrelated individuals using relatedness estimated from genotype data [60]. Under a univariate polygenic model, the phenotypic variance (*σ^2^_p_*) of a trait is decomposed into genetic (*σ^2^_g_*) and environmental (*σ^2^_e_*) components. Environmental variance incapsulates all variance that is not genetic, including error. Narrow-sense heritability (*h^2^*) is the proportion of phenotypic variance accounted for by additive genetic variance (*h^2^* = *σ^2^_g_*/*σ^2^_p_*). To determine whether heritability (*h^2^*) was significantly greater than 0, likelihood of the polygenic model was compared to that of a model with *h^2^* constrained to 0. Under a bivariate polygenic model, phenotypic covariance between two traits is decomposed into genetic and environmental components to determine the extent to which traits are influenced by shared genetic effects. Since genetic correlations between traits are only meaningful if traits are heritable, we estimated heritability of all traits. Bivariate polygenic analyses were then applied to significantly heritable pairs of traits to estimate genetic (*ρ_g_*), environmental (*ρ_e_*), and phenotypic (*ρ_p_)* correlations. The genetic correlation (*ρ_g_*) denotes the correlation between latent additive genetic factors influencing both traits. The environmental correlation (*ρ_e_*) denotes the correlation between nongenetic factors influencing both traits. To determine whether genetic (*ρ_g_*) and environmental (*ρ_e_*) correlations were significantly different from 0, likelihood of the bivariate polygenic model was compared to that of a model where the parameter of interest was constrained to 0.

### Gene × Age interaction models

A polygenic model can be extended to examine Gene × Environment (G × E) interactions [36–38]. One consequence of G × E is that additive genetic variance is greater under certain environments than others. To test for this effect with a continuous environmental variable (age), the polygenic model is modified to include a linear function on the logarithm of *σ^2^_g_.* This linear function contains a free parameter, *γ*, reflecting change in *σ^2^_g_* unit of the environmental variable (age in years). A nonzero value of *γ* implies a heritable response to the environment, and therefore, a G × E interaction. This G × E interaction tests for fluctuations (with age) in action of genetic factors and a significant G × E interaction suggests a change in magnitude of effect of specific genetic factors (with age). A second consequence of G × E is that the trait exhibits imperfect pleiotropy with itself, that is, the genetic correlation between the trait measured under one environment and the trait measured under another environment is less than 1. This phenomenon can be examined in cross-sectional studies where individuals are tested under a single environment (timepoint), provided relatedness between individuals is known [37]. To test for this effect, the polygenic model is modified to include another free parameter, *λ*, reflecting the rate of decay in genetic correlation (*ρ_g_*) as difference in the environmental variable increases. A nonzero value of *λ* implies imperfect pleiotropy, and therefore, a G × E interaction. This G × E interaction tests for variation in genetic factors influencing the trait (at different ages) and a significant G × E interaction suggests a change (with age) in the genetic factors themselves. G × E interaction models were fitted to heritable traits, with age in years as the continuous environmental variable that is, Gene × Age interactions. See Table S3 for more information.

All models included age, age^2^, sex, and their interactions as covariates. To adjust for multiple testing, false discovery rate (FDR) was set at 5% [61]. Rank-based inverse normal transformations were applied to all traits to ensure normality.

### Sensitivity analyses

In addition to the bifactor model described above, we applied a confirmatory hierarchical model (Figure 1b) in Mplus [52] to generate four correlated factors of psychopathology and a general factor. We generated the same factors as the bifactor model: specific factors of (a) anxious-misery, (b) externalizing, (c) fear, and (d) psychosis-spectrum, and (e) a general factor. In bifactor models, general and specific factors are orthogonal, whereas in hierarchical models, the general factor is defined by the specific factors and thus general and specific factors are explicitly correlated. Univariate, bivariate, and G × A analyses, as described above, were repeated on these factors to examine the contaminating effect, that is shared variance of general and specific factors. Table S4 shows factor loadings, Table S5 shows correlations between factors (and with cognition), Figure S2 shows test information plots.

## Results

### Externalizing psychopathology is heritable

**Figure 2.**
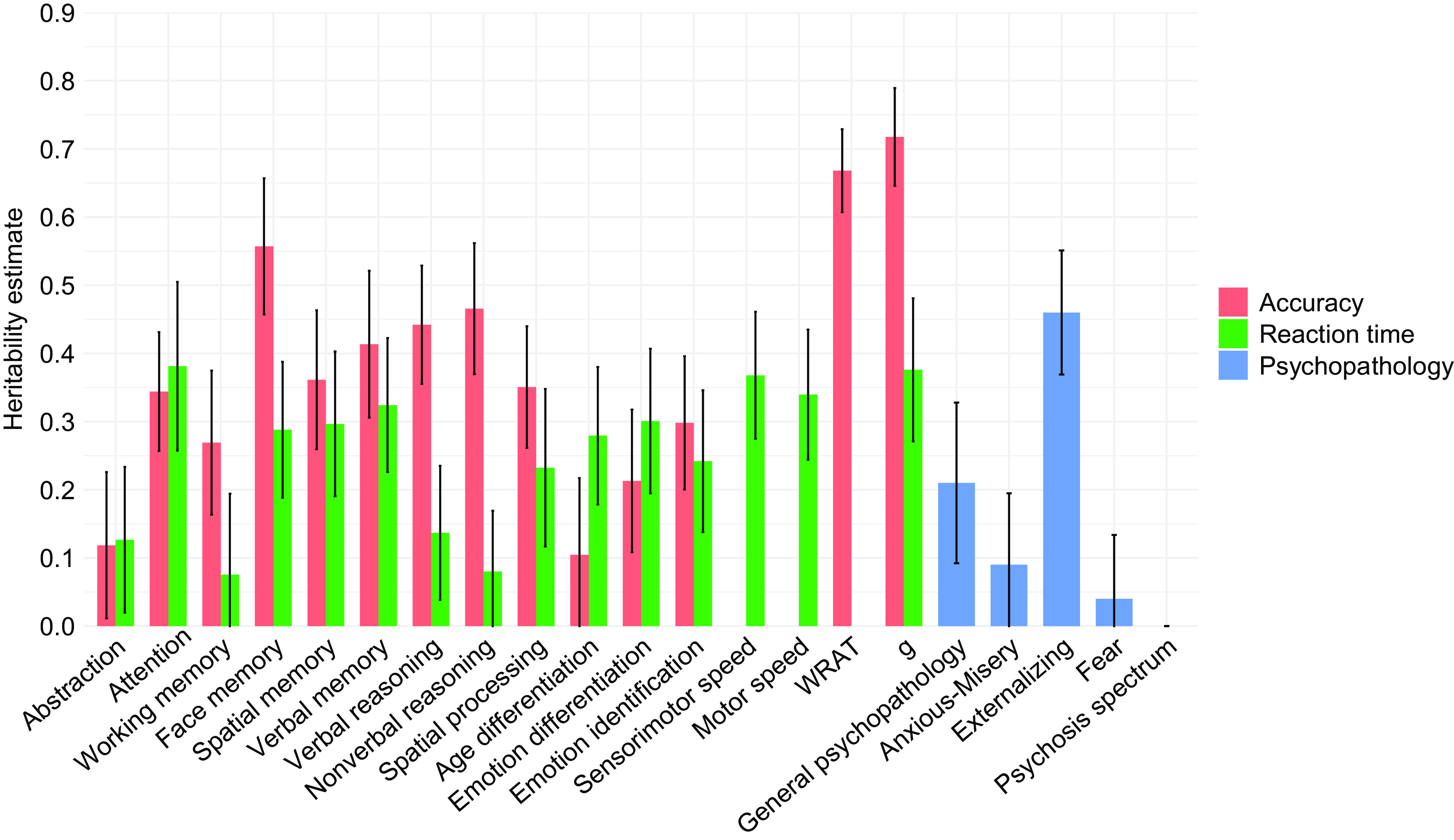
Heritability estimates for all neurocognitive measures and psychopathology factors. *Error bars represent standard errors (SEs).

Significant heritability estimates were observed for general (*h^2^ =* 0.21*, p =* 0.040*)* and externalizing psychopathology (*h^2^ =* 0.46*, p =* 2 × 10^−6^), but only externalizing survived FDR correction (Figure 2 and Table 1). As reported previously [23], most accuracy (*h^2^* range = 0.21–0.72) and reaction time (*h^2^* range = 0.23–0.38) measures were also heritable. Subsequent bivariate and G × A analyses were run on significantly heritable traits (after FDR correction).

**Table 1.** Heritability estimates for all traits, genetic, and phenotypic correlations between externalizing and cognition.

Category	Trait	*h^2^*	*p*	SE		Externalizing				
					*ρg*	*p*	SE	*ρp*	*p*	SE
Accuracy	Abstraction	0.12	0.143	0.107	–	–	–	–	–	–
	Attention	**0.34**	**5 × 10**^**−5**^	**0.087**	0.066	0.689	0.166	**−0.071**	**1 × 10**^**−6**^	**0.015**
	Working memory	**0.27**	**0.006**	**0.106**	−0.387	0.067	0.229	**−0.069**	**2 × 10**^**−6**^	**0.015**
	Face memory	**0.56**	**1 × 10**^**−6**^	**0.100**	**−0.412**	**0.004**	**0.165**	**−0.049**	**0.001**	**0.015**
	Spatial memory	**0.36**	**0.001**	**0.102**	−0.134	0.434	0.175	**−0.044**	**0.003**	**0.015**
	Verbal memory	**0.41**	**4 × 10**^**−4**^	**0.108**	−0.180	0.274	0.171	−0.028	0.059	0.015
	Verbal reasoning	**0.44**	**3 × 10**^**−6**^	**0.087**	**−0.485**	**0.001**	**0.159**	**−0.111**	**4 × 10**^**−14**^	**0.015**
	Nonverbal reasoning	**0.47**	**1 × 10**^**−5**^	**0.096**	−0.338	0.019	0.149	**−0.065**	**1 × 10**^**−5**^	**0.015**
	Spatial reasoning	**0.35**	**2 × 10**^**−4**^	**0.089**	**−0.426**	**0.010**	**0.175**	**−0.093**	**2 × 10**^**−10**^	**0.015**
	Age differentiation	0.10	0.184	0.112	–	–	–	–	–	–
	Emotion differentiation	**0.21**	**0.024**	**0.105**	−0.208	0.371	0.237	**−0.042**	**0.004**	**0.015**
	Emotion identification	**0.30**	**0.002**	**0.098**	0.052	0.778	0.188	−0.023	0.120	0.015
	WRAT	**0.67**	**2 × 10**^**−21**^	**0.061**	**−0.314**	**0.002**	**0.109**	**−0.127**	**5 × 10**^**−18**^	**0.015**
	Composite score (*g*)	**0.72**	**2 × 10**^**−14**^	**0.072**	**−0.394**	**2 × 10**^**−4**^	**0.118**	**−0.109**	**1 × 10**^**−13**^	**0.015**
Reaction time	Abstraction	0.13	0.120	0.107	–	–	–	–	–	–
	Attention	**0.38**	**0.002**	**0.124**	0.123	0.492	0.183	0.003	0.831	0.015
	Working memory	0.08	0.263	0.119	–	–	–	–	–	–
	Face memory	**0.29**	**0.003**	**0.100**	0.038	0.848	0.202	**−0.048**	**0.001**	**0.015**
	Spatial memory	**0.30**	**0.004**	**0.106**	0.115	0.570	0.208	**−0.066**	**6 × 10**^**−6**^	**0.015**
	Verbal memory	**0.32**	**5 × 10**^**−4**^	**0.098**	0.292	0.137	0.219	−0.009	0.541	0.015
	Verbal reasoning	0.14	0.094	0.098	–	–	–	–	–	–
	Nonverbal reasoning	0.08	0.184	0.089	–	–	–	–	–	–
	Spatial reasoning	**0.23**	**0.029**	**0.116**	−0.034	0.880	0.222	**−0.066**	**6 × 10**^**−6**^	**0.015**
	Age differentiation	**0.28**	**0.004**	**0.101**	0.185	0.338	0.201	**−0.055**	**2 × 10**^**−4**^	**0.015**
	Emotion differentiation	**0.30**	**0.004**	**0.106**	0.184	0.337	0.198	−0.005	0.752	0.015
	Emotion identification	**0.24**	**0.011**	**0.104**	0.459	0.032	0.247	**0.036**	**0.013**	**0.015**
	Sensorimotor speed	**0.37**	**1 × 10**^**−4**^	**0.093**	−0.155	0.361	0.183	0.019	0.205	0.015
	Motor speed	**0.34**	**0.001**	**0.095**	**−0.659**	**1 × 10**^**−4**^	**0.222**	**−0.041**	**0.005**	**0.015**
	Speed composite score (*gs*)	**0.38**	**5 × 10**^**−4**^	**0.105**	−0.194	0.259	0.201	0.026	0.080	0.015
Psychopathology	General psychopathology	0.21	0.040	0.118	–	–	–	–	–	–
	Anxious—misery	0.09	0.183	0.105	–	–	–	–	–	–
	externalizing	**0.46**	**2 × 10**^**−6**^	**0.091**	–	–	–	–	–	–
	Fear	0.04	0.337	0.094	–	–	–	–	–	–
	Psychosis	0.00	0.494	0.000	–	–	–	–	–	–

Bolded estimates significant after correction for multiple testing (FDR = 0.05).

Abbreviations: AWRAT, Wide Range Achievement Test; SE, standard error.

### Overlapping genetic factors on externalizing and cognition suggest pleiotropic effects

Externalizing showed significant negative phenotypic correlations with most accuracy measures (range *ρ_p_* = −0.042 to −0.127) and some reaction time measures (range *ρ_p_* = −0.036 to −0.66; Table 1) after FDR correction, such that greater psychopathology was associated with poorer accuracy and slower reaction times. Significant negative genetic correlations were observed after FDR correction between externalizing and accuracy measures of face memory (*ρ_g_* = −0.412, *p* = 0.004), verbal reasoning (*ρ_g_* = −0.485, *p* = 0.001), spatial processing (*ρ_g_* = −0.426, *p* = 0.010), general verbal knowledge (*ρ_g_* = −0.314, *p* = 0.002), *g* (*ρ_g_* = −0.44, *p* = 0.002), and sensorimotor speed (*ρ_g_* = −0.659, *p* = 1 × 10^−4^) suggesting that genetic factors underlying externalizing overlap with lower accuracy and slower reaction times.

### Genetic variance on externalizing decreases with age

**Figure 3.**
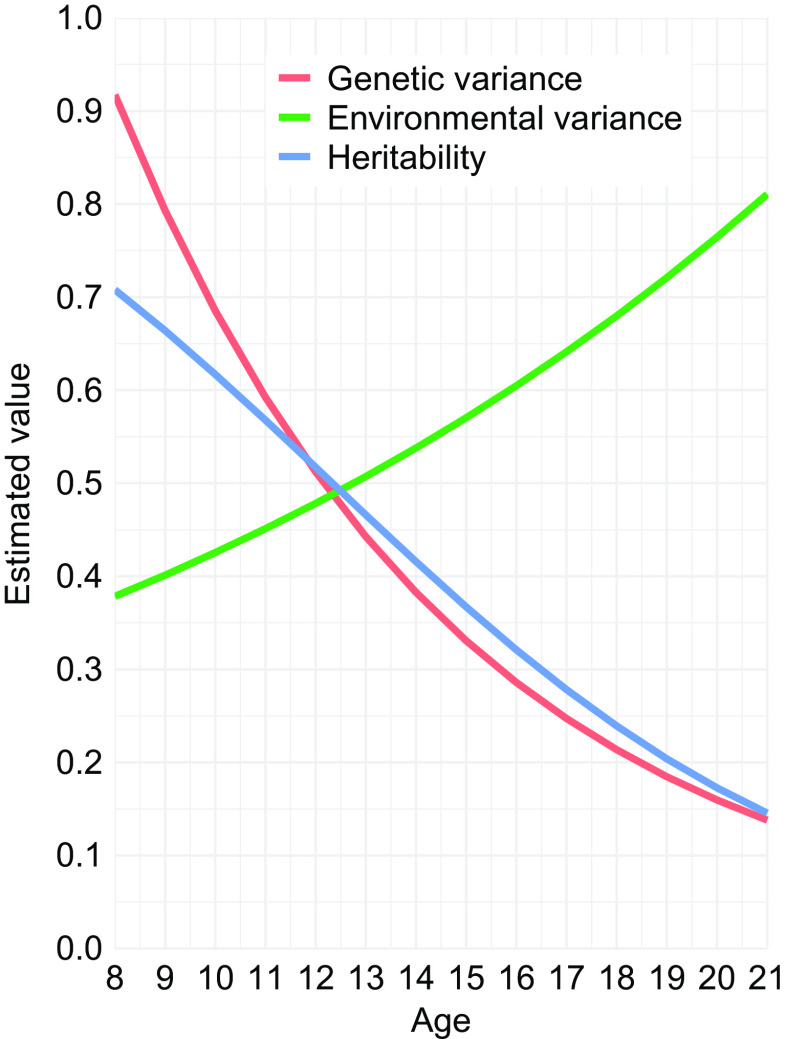
Estimated genetic variance, environmental variance and heritability by age for externalizing.

Significant decrease in genetic variance was observed on externalizing (*γ_g_* = −0.146, *p* = 0.004; Figure 3), suggesting that specific genetic factors influence externalizing psychopathology between childhood and early adulthood, but also that the magnitude of effect of these genetic factors decreases with age. Significant increase in environmental variance was also observed (*γ_e_* = 0.059, *p* = 0.009). Decay in genetic correlation did not reach statistical significance (*λ* = 0.027, *p* = 0.423), such that we did not find evidence for change in genetic factors, that is, novel genetic influences. Data presented in Figure 3 were generated using Formula 5 in Table S3.

### Results of genetic analyses are robust to factor analytic approach

Univariate, bivariate, and G × A analyses conducted on psychopathology factors derived from the hierarchical model generated similar results. Externalizing (*h^2^ =* 0.58*, p =* 1x10^−7^) remained heritable, but general psychopathology (*h^2^ =* 0.37*, p =* 0.001) anxious-misery (*h^2^ =* 0.35*, p =* 0.002) and fear (*h^2^ =* 0.25*, p =* 0.011) were also significantly heritable, although fear did not survive FDR correction (Table S6 and Figure S3). General psychopathology and anxious-misery showed significant phenotypic correlations with cognition (Table S6), but only externalizing showed significant genetic correlations with verbal reasoning (*ρ_g_* = −0.43, *p* = 0.001), general verbal knowledge (*ρ_g_* = −0.25, *p* = 0.005), and *g (ρ_g_* = −0.37, *p* = 3x10^−6^), after FDR correction. Again, significant decrease in genetic variance (*γ_g_* = −0.112, *p* = 0.029) and increase in environmental variance (*γ_e_* = 0.089, *p* = 0.008) were observed for externalizing (Figure S4) Change in genetic variance on general psychopathology (*γ_g_* = 0.017, *p* = 0.279) and anxious-misery (*γ_g_* = 0.023, *p* = 0.269) was not significant. Data presented in Figure S4 were generated using Formula 5 in Table S3.

## Discussion

Using a large, population-based cohort of individuals aged 8–21, we showed that externalizing psychopathology in the first two decades of life is under considerable genetic influence. Externalizing showed genetic overlap with lower performance on face memory, verbal reasoning, spatial processing, motor speed, verbal knowledge, and general cognitive ability. We did not find evidence for novel genetic factors on externalizing throughout this developmental period, rather we found a decrease in genetic variance, and increase in environmental variance. These findings have several implications for our understanding of early life psychopathology.

First, to the best of our knowledge, this is the first study to use a large, population-based cohort and genetic relatedness matrix to estimate heritability of data-driven factors of psychopathology (both orthogonal and correlated) throughout childhood and early adulthood. Our finding of heritable general psychopathology, anxious-misery, externalizing, and fear are in line with previous evidence [11–15]. We did not find evidence for genetic influences on the psychosis-spectrum factor, in contrast to prior evidence [11,12]. However, SNP heritability estimates of psychotic symptoms are more modest, with a report of nonsignificant and zero estimates for paranoia and hallucinations, respectively [62]. Moreover, while there has been progress in delineating molecular genetic underpinnings of adult schizophrenia [63], the same cannot be said of childhood psychotic symptoms [64]. Notably, subjects were less likely to endorse items that loaded on the psychosis-spectrum factor (average 11% endorsing), than on externalizing (21%), anxious-misery (15%), and fear (16%). Similarly, clinical diagnostic rates of attention deficit, oppositional defiant, and conduct disorders in PNC are 16, 33, and 7%, respectively [40], while rate of threshold psychotic symptoms is 4% [65]. Psychotic symptoms also emerge later during development than symptoms relating to externalizing, anxious-misery, and fear [66,67].

In contrast, we found externalizing psychopathology to be significantly and moderately heritable, with genetic factors explaining 46–58% of the variance. Equally substantial genetic influences on externalizing between ages 8 and 26 have been reported in twin and adoption studies [13–15]. A very similar SNP heritability of 0.44 has also been reported for externalizing [68], but another study found SNP heritability estimates of zero for a range of externalizing problems [69]. Differences between SNP and pedigree heritability estimates are likely due to rare variants not well captured by common SNPs, with SNP heritability representing the lower bound. Thus, using related individuals is more powerful than using unrelated individuals when estimating heritability, and the combination of all possible relationships, as in our sample, results in something of a hybrid between SNP and pedigree estimates. However, shared environment may contribute to heritability inflation when using related individuals. Nevertheless, rare variants account for a significant proportion of total heritability [70], and our heritability estimates are generally in line with previous studies.

Importantly, we found significant heritability estimates for general psychopathology and anxious-misery factors generated from a hierarchical, but not bifactor, model. This finding highlights the utility of bifactor models, in which all factors (both general and specific) are orthogonal [40,71], thereby allowing us to examine the unique contribution of genetic factors to general psychopathology and specific factors of anxious-misery, externalizing, fear, and psychosis-spectrum [43,44]. This finding, as well as the finding of stronger genetic influences on externalizing than general psychopathology, is also in line with previous evidence of genetic signal on specific cognitive factors emerging only when variance associated with general cognitive ability is parsed out, that is when applying a bifactor model [71]. In hierarchical models, on the other hand, variance associated with the general factor may account for significant findings in the specific factors, and vice versa. Thus, our findings suggest that genetic effects underlying early-life anxiety and depression may underlie more general psychopathology. Estimates of genetic influence on childhood anxiety [17,18], depression [19,20], and phobias [72,73] are heterogeneous, ranging from small to large (*h*^2^ = 0.15–0.77) for anxiety and depression [17,19], and for phobias (*h*^2^ = 0.23–0.72) [72,73].

Second, we found negative genetic correlations between externalizing and face memory, verbal reasoning, spatial processing, sensorimotor speed, verbal knowledge, and general cognitive ability (*g*). These findings are in line with previous evidence of genetic overlap between ADHD and general and executive cognitive functions [27–32,35], but expand knowledge by showing that genetic overlap extends to externalizing psychopathology more generally, as well as to memory, complex reasoning, and speed functions. Similarly, in the same sample, genetic overlap was reported between inattention and memory, social cognition, executive function, and complex reasoning [74]. Genetic overlap between externalizing and cognitive functioning may be due to the same genetic factors influencing both traits. A recent genome-wide meta-analyses identified shared risk loci for ADHD and intelligence [31]. Rare genetic variants have also been identified for cognition [75,76] and ADHD [77], with overlapping genes implicated in ADHD studies of common and rare variants [78]. However, while there is evidence of common and rare variants disrupting similar biological pathways in ADHD [79], the neurobiological mechanisms underlying shared genetic influences on ADHD and intelligence remain unclear [31]. Future studies that utilize animal models are needed to elucidate the causal and biological pathways underlying these shared genetic influences. Alternatively, cognitive impairments may lead to externalizing, and/or vice versa. Importantly, different cognitive measures show different psychometric properties and associations with specific functions require replication. Future longitudinal studies incorporating behavioral, neuroimaging, and genetic data can further disentangle these associations. Nevertheless, our findings suggest that cognitive impairment may be a useful endophenotype [80,81] of externalizing psychopathology. Interestingly, the WRAT, a measure of verbal knowledge showed the strongest phenotypic correlation with externalizing, but the weakest (statistically significant) genetic correlation. This finding highlights the importance of elucidating genetic underpinnings of phenotypic associations to delineate biological etiology.

Finally, we found static and dynamic genetic influences on externalizing psychopathology between childhood and early adulthood, in line with previous evidence [13–15,82]. While we did not find evidence for novel genetic influences, we found a decrease in genetic variance, and increase in environmental variance. Our findings are in line with those of Huizink [15], who found a decrease in genetic influences on externalizing from 43 to 29% between age 12 and 26, as well as an increase in environmental influences from 39 to 52% between these ages. Similarly, Wichers [14] found a decrease in genetic effects from 78 to 73% and an increase in environmental effects from 20 to 26% between age 8 and 20, but also reported novel genetic influences throughout adolescence [14]. Huizink, on the other hand, reported novel environmental, but not genetic, influences [15]. Several phenomena may underlie these G × A interactions. Genes may become less expressed due to maturational processes involving hormonal and physical development [13]. Increasing environmental influences likely reflect growing peer influences and substance use [83]. Of note, environmental variance in this study incapsulates all variance that is not genetic. However, measurement error is unlikely to account for our findings since reliability of our factors is high (Figures S1 and S2). Moreover, method of symptom reporting differed by age, and genetic effects may differ by reporting method. For example, Scourfield et al. [84] found heritability estimates of 54 and 35% for parent- and self-reported conduct problems, respectively. Nevertheless, G × A interaction analyses adjusting additionally for reporting method generated similar findings, with significant decrease in genetic variance (*γ_g_* = −0.151, *p* = 0.002; *γ_g_* = −0.112, *p* = 0.026), and increase in environmental variance (*γ_e_* = 0.058, *p* = 0.006; *γ_e_* = 0.092, *p* = 0.007) on externalizing factors derived from bifactor and hierarchical models, respectively. Future studies that are able to combine longitudinal, self- and parent-report symptom data will help elucidate these age-associated effects further. Conversely, we previously reported increasing genetic and environmental variance on general cognitive ability in this sample [23]. Thus, while a proportion of genetic factors underlying externalizing and cognition overlap, other, nonoverlapping genetic influences may show diverging developmental trajectories. Advanced quantitative genetic methods may shed light on trajectories of shared genetic influences.

This study has limitations. First, our analyses were restricted to European American individuals and future studies should include other populations. Second, our data were cross-sectional and longitudinal studies with repeated assessments of identical measures and individuals are needed to fully establish age-related changes in genetic factors. Third, lower heritability estimates for general psychopathology and anxious-misery meant less power to detect genetic correlations with cognition and G × A interactions. Finally, although we used a large sample, and comprehensive assessments of cognition and psychopathology, our findings require replication.

## Data Availability

The data that support the findings of this study are openly available in the database of Genotypes and Phenotypes (dbGaP): https://www.ncbi.nlm.nih.gov/projects/gap/cgi-bin/study.cgi?study_id=phs000607.v3.p2.

## Supplementary material

For supplementary material accompanying this paper visit https://doi.org/10.1192/j.eurpsy.2021.21. click here to view supplementary material
