# Supplementary material for: Genetic influences on externalizing psychopathology overlap with cognitive functioning and show developmental variation
Source: Eur Psychiatry. 2021 Mar 31;64(1):e29. doi: 10.1192/j.eurpsy.2021.21 (PMC8080212; doi:10.1192/j.eurpsy.2021.21)
Supplement: Supplementary file 1 [file S0924933821000213sup001.docx]

| **Table S1** Factor loadings for the confirmatory bifactor model of 112 items from the GOASSESS structured interview | | | | | | |
| --- | --- | --- | --- | --- | --- | --- |
| Item* | Question | General | Anxious-Misery | Externalizing | Fear | Psychosis- Spectrum |
| GAD002 | Do you tend to worry a lot more than most children/people your age? | 0.393 | 0.751 | 0 | 0 | 0 |
| GAD001 | Have you ever been a worrier? | 0.369 | 0.727 | 0 | 0 | 0 |
| OCD007 | Have you ever been bothered by thoughts that don't make sense to you, that come over and over again and won't go away, such as need for symmetry/exactness? | 0.457 | 0.524 | 0 | 0 | 0 |
| OCD016 | Have you ever had to do something over and over again - that would have made you feel really nervous if you couldn't do it, like ordering or arranging things? | 0.543 | 0.498 | 0 | 0 | 0 |
| DEP001 | Has there ever been a time when you felt sad or depressed most of the time? | 0.627 | 0.478 | 0 | 0 | 0 |
| OCD011 | Have you ever had to do something over and over again - that would have made you feel really nervous if you couldn't do it, like cleaning or washing (for example, your hands, house)? | 0.540 | 0.437 | 0 | 0 | 0 |
| PAN001 | Have you ever had a panic attack? | 0.480 | 0.437 | 0 | 0 | 0 |
| DEP002 | Has there ever been a time when you cried a lot, or felt like crying? | 0.616 | 0.422 | 0 | 0 | 0 |
| OCD003 | Have you ever been bothered by thoughts that don't make sense to you, that come over and over again and won't go away, such as thoughts about contamination/germs/illness? | 0.528 | 0.416 | 0 | 0 | 0 |
| OCD012 | Have you ever had to do something over and over again - that would have made you feel really nervous if you couldn't do it, like counting? | 0.541 | 0.392 | 0 | 0 | 0 |
| SUI002 | Have you ever thought about killing yourself? | 0.585 | 0.366 | 0 | 0 | 0 |
| OCD019 | Do you feel the need to do things just right (like they have to be perfect)? | 0.470 | 0.356 | 0 | 0 | 0 |
| PAN004 | Has there ever been a time when all of a sudden, you felt that you were losing control, something terrible was going to happen, that you were going crazy, or going to die? | 0.637 | 0.351 | 0 | 0 | 0 |
| PAN003 | Has there ever been a time when all of a sudden you felt very, very scared or uncomfortable - and your chest hurt, you couldn't catch your breath, your heart beat very fast, you felt very shaky, and sweaty/tingly/numb in your hands or feet? | 0.577 | 0.348 | 0 | 0 | 0 |
| OCD001 | Have you ever been bothered by thoughts that don't make sense to you, that come over and over again and won't go away, such as concern with harming others or yourself? | 0.707 | 0.321 | 0 | 0 | 0 |
| OCD013 | Have you ever had to do something over and over again - that would have made you feel really nervous if you couldn't do it, like checking (for example, doors, locks, ovens)? | 0.589 | 0.320 | 0 | 0 | 0 |
| OCD017 | Have you ever had to do something over and over again - that would have made you feel really nervous if you couldn't do it, like doing things over and over again at bedtime, like arranging the pillows, sheets, or other things? | 0.598 | 0.306 | 0 | 0 | 0 |
| SIP032 | Do you ever feel a loss of sense of self or feel disconnected from yourself or your life? | 0.602 | 0.302 | 0 | 0 | 0 |
| OCD005 | Have you ever been bothered by thoughts that don't make sense to you, that come over and over again and won't go away, such as feeling that bad things that happened were your fault? | 0.722 | 0.293 | 0 | 0 | 0 |
| SCR007 | Have you ever had to go to a hospital and stay overnight because of problems with your mood, feelings, or how you were acting? | 0.475 | 0.275 | 0 | 0 | 0 |
| SUI001 | Have you ever thought a lot about death or dying? | 0.560 | 0.266 | 0 | 0 | 0 |
| OCD008 | Have you ever been bothered by thoughts that don't make sense to you, that come over and over again and won't go away, such as religious thoughts? | 0.620 | 0.240 | 0 | 0 | 0 |
| OCD014 | Have you ever had to do something over and over again - that would have made you feel really nervous if you couldn't do it, like getting dressed over and over again? | 0.626 | 0.232 | 0 | 0 | 0 |
| DEP004 | Has there ever been a time when you felt grouchy, irritable, or in a bad mood most of the time even little things would make you mad? | 0.742 | 0.231 | 0 | 0 | 0 |
| DEP006 | Has there ever been a time when nothing was fun for you and you just weren't interested in anything? | 0.705 | 0.231 | 0 | 0 | 0 |
| OCD004 | Have you ever been bothered by thoughts that don't make sense to you, that come over and over again and won't go away, such as fear that you would do something/say something bad without intending to? | 0.753 | 0.229 | 0 | 0 | 0 |
| OCD018 | Have you ever saved up so many things that people complained or they got in the way? | 0.498 | 0.228 | 0 | 0 | 0 |
| SCR001 | Have you ever talked to a counselor, psychologist, social worker, psychiatrist or some other professional about your feelings or problems with your mood or behaviors? | 0.463 | 0.226 | 0 | 0 | 0 |
| OCD015 | Have you ever had to do something over and over again - that would have made you feel really nervous if you couldn't do it, like going in and out a door over and over again? | 0.591 | 0.214 | 0 | 0 | 0 |
| OCD006 | Have you ever been bothered by thoughts that don't make sense to you, that come over and over again and won't go away, such as forbidden/bad thoughts? | 0.745 | 0.209 | 0 | 0 | 0 |
| OCD002 | Have you ever been bothered by thoughts that don't make sense to you, that come over and over again and won't go away, such as pictures of violent things? | 0.710 | 0.190 | 0 | 0 | 0 |
| SEP510 | Did you ever worry/have bad dreams about something terrible happening to you or your mother/father/guardian so that you would not see them again? | 0.517 | 0.123 | 0 | 0 | 0 |
| SIP033 | Has anyone pointed out to you that you are less emotional or connected to people than you used to be? | 0.583 | 0.089 | 0 | 0 | 0 |
| SIP039 | Within the past 6 months, are you having a harder time getting normal activities done? | 0.467 | 0.085 | 0 | 0 | 0 |
| MAN007 | Has there ever been a time when you felt unusually grouchy, cranky, or irritable; when the smallest things would make you really mad? | 0.769 | 0.074 | 0 | 0 | 0 |
| ADD012 | Have you often had problems following instructions and often fail to finish school, work, or other things you meant to get done? | 0.410 | 0 | 0.759 | 0 | 0 |
| ADD011 | Have you often had trouble paying attention or keeping your mind on your school, work, chores, or other activities that you were doing? | 0.452 | 0 | 0.756 | 0 | 0 |
| ADD016 | Have you often had people tell you that you did not seem to be listening when they spoke to you or that you were daydreaming? | 0.495 | 0 | 0.655 | 0 | 0 |
| ODD002 | Was there a time when you often got into trouble with adults for refusing to do what they told you to do or for breaking rules at home/school/work? | 0.518 | 0 | 0.649 | 0 | 0 |
| ADD021 | Have you often blurted out answers to other people's questions before they finished speaking or interrupted people abruptly? | 0.382 | 0 | 0.634 | 0 | 0 |
| ADD022 | Have you often joined other people's conversations or had trouble waiting your turn (e.g., waiting in line, waiting for a teacher to call on you in class)? | 0.413 | 0 | 0.618 | 0 | 0 |
| ODD001 | Was there a time when you often did things that got you into trouble with adults such as losing your temper, arguing with or talking back to adults, or being grouchy or irritable with them? | 0.535 | 0 | 0.604 | 0 | 0 |
| ODD003 | Did you often annoy other people on purpose or blame other people for your mistakes (excluding siblings)? | 0.441 | 0 | 0.602 | 0 | 0 |
| ADD013 | Have you often disliked, avoided, or put off school or homework (or any other activity requiring concentration) | 0.445 | 0 | 0.596 | 0 | 0 |
| ADD014 | Have you often lost things you needed for school or projects at home (assignments or books) or made careless mistakes in school work or other activities? | 0.416 | 0 | 0.594 | 0 | 0 |
| ADD015 | Have you often had trouble making plans, doing things that had to be done in a certain kind of order or that had a lot of different steps? | 0.470 | 0 | 0.534 | 0 | 0 |
| ADD020 | Have you often had difficulty sitting still for more than a few minutes at a time, even after being asked to stay seated, or have you often fidgeted with your hands or feet or wiggled in your seat? | 0.491 | 0 | 0.524 | 0 | 0 |
| ODD005 | Did you ever get into trouble for getting even with other people by doing things to hurt them, telling lies about them, or messing up their things? | 0.468 | 0 | 0.520 | 0 | 0 |
| CDD005 | Did you often bully others (hitting, threatening or scaring someone who was younger or smaller), threaten or frighten someone on purpose, or often start physical fights with others? | 0.470 | 0 | 0.514 | 0 | 0 |
| CDD003 | Did you ever set fires, break into cars, or destroy someone else's property on purpose? | 0.459 | 0 | 0.489 | 0 | 0 |
| ODD006 | Were you often irritable or grouchy, or did you often get angry because you thought that things were unfair? | 0.612 | 0 | 0.485 | 0 | 0 |
| CDD001 | Was there ever a time when you often did things that got you into trouble with adults like lying or stealing (something worth more than $5) from family, others, or stores? | 0.443 | 0 | 0.442 | 0 | 0 |
| CDD008 | Have you ever threatened someone? | 0.546 | 0 | 0.415 | 0 | 0 |
| CDD006 | Have you ever been physically cruel to an animal or person (on purpose)? | 0.462 | 0 | 0.414 | 0 | 0 |
| CDD007 | Have you ever tried to hurt someone with a weapon (a bat, brick, broken bottle, knife, or gun)? | 0.491 | 0 | 0.410 | 0 | 0 |
| CDD004 | Do you have a probation officer or have you ever been on probation? | 0.305 | 0 | 0.346 | 0 | 0 |
| SCR006 | Are you currently taking medication because of your emotions and/or behaviors? | 0.376 | 0 | 0.331 | 0 | 0 |
| CDD002 | Have you ever skipped school, stayed out at night later than you were supposed to (more than 2 hours), or run away from home overnight? | 0.446 | 0 | 0.300 | 0 | 0 |
| SCR008 | Have you or anyone else (like your friends, parents, or teachers) ever thought you needed help because of problems with your mood, feelings, or how you were acting? | 0.530 | 0 | 0.223 | 0 | 0 |
| SIP038 | Within the past 6 months, are you having a harder time getting your work or schoolwork done? | 0.394 | 0 | 0.174 | 0 | 0 |
| SOC003 | Was there ever a time in your life when you felt afraid or uncomfortable when you had to do something in front of a group of people, like speaking in class? | 0.360 | 0 | 0 | 0.694 | 0 |
| SOC004 | Was there ever a time in your life when you felt afraid or uncomfortable acting, performing, giving a talk/speech, playing a sport or doing a musical performance, or taking an important test or exam (even though you studied enough)? | 0.433 | 0 | 0 | 0.680 | 0 |
| SOC005 | Was there ever a time in your life when you felt afraid or uncomfortable because you were the center of attention and were concerned something embarrassing might happen and you felt very afraid or felt uncomfortable? | 0.469 | 0 | 0 | 0.646 | 0 |
| SOC001 | Was there ever a time in your life when you felt afraid or uncomfortable or really, really shy with people, like meeting new people, going to parties, or eating or drinking, writing or doing homework in front of others? | 0.421 | 0 | 0 | 0.617 | 0 |
| SOC002 | Was there ever a time in your life when you felt afraid or uncomfortable talking on the telephone or with people your own age who you don't know very well? | 0.399 | 0 | 0 | 0.541 | 0 |
| AGR005 | Have you ever been very nervous or afraid of traveling by yourself? | 0.491 | 0 | 0 | 0.520 | 0 |
| AGR002 | Have you ever been very nervous or afraid of going to public places (such as a store or shopping mall)? | 0.552 | 0 | 0 | 0.510 | 0 |
| AGR006 | Have you ever been very nervous or afraid of traveling away from home? | 0.548 | 0 | 0 | 0.508 | 0 |
| AGR001 | Have you ever been very nervous or afraid of being in crowds (for example, a classroom, cafeteria, restaurant, or movie theater)? | 0.551 | 0 | 0 | 0.484 | 0 |
| AGR008 | Have you ever been very nervous or afraid of using public transportation like a bus, train, or subway? | 0.559 | 0 | 0 | 0.465 | 0 |
| AGR004 | Have you ever been very nervous or afraid of going over bridges or through tunnels? | 0.507 | 0 | 0 | 0.435 | 0 |
| PHB007 | Have you ever been very nervous or afraid of flying or airplanes? | 0.390 | 0 | 0 | 0.409 | 0 |
| AGR003 | Have you ever been very nervous or afraid of being in an open field? | 0.551 | 0 | 0 | 0.403 | 0 |
| PHB004 | Have you ever been very nervous or afraid of storms, thunder, or lightning? | 0.276 | 0 | 0 | 0.383 | 0 |
| PHB002 | Have you ever been very nervous or afraid of being in really high places, like a roof or tall building? | 0.340 | 0 | 0 | 0.370 | 0 |
| PHB001 | Have you ever been very nervous or afraid of animals or bugs, like dogs, snakes, or spiders? | 0.283 | 0 | 0 | 0.356 | 0 |
| PHB006 | Have you ever been very nervous or afraid of closed spaces, like elevators or closets? | 0.412 | 0 | 0 | 0.331 | 0 |
| SEP509 | When you knew that you were going to be away from home or mother/father/guardian, have you ever become very upset and worried (e.g., when you learned your mother/father/guardian were going on an upcoming trip or night out)? | 0.400 | 0 | 0 | 0.304 | 0 |
| AGR007 | Have you ever been very nervous or afraid of traveling in a car? | 0.471 | 0 | 0 | 0.290 | 0 |
| PHB005 | Have you ever been very nervous or afraid of doctors, needles, or blood? | 0.203 | 0 | 0 | 0.279 | 0 |
| SEP508 | Has there ever been a time when you wanted to stay home from school or not go to other places without your mother, father, or guardian? | 0.374 | 0 | 0 | 0.272 | 0 |
| PHB003 | Have you ever been very nervous or afraid of water or situations involving water, such as a swimming pool, lake, or ocean? | 0.383 | 0 | 0 | 0.265 | 0 |
| PHB008 | Have you ever been very nervous or afraid of specific things or situations? | 0.360 | 0 | 0 | 0.229 | 0 |
| SEP500 | Since you were 5 years old, has there ever been a time when you had a lot of worries about your attachment figures (mother, father, guardian) and were very upset or got sick (for example, felt sick to your stomach, headaches, thrown-up) when you were away from them? | 0.419 | 0 | 0 | 0.223 | 0 |
| SEP511 | Have you ever been scared to be alone in your room (or any place in your house) or did you need your mother/father/guardian to stay with you while you fell asleep? | 0.279 | 0 | 0 | 0.147 | 0 |
| SIP028 | Do people ever seem to have difficulty understanding you? | 0.476 | 0 | 0 | 0 | 0.658 |
| SIP027 | Do people ever tell you that they can't understand you? | 0.458 | 0 | 0 | 0 | 0.635 |
| SIP010 | Based on your experiences within the past year, please tell me how much you agree or disagree with the following statements: I believe that I have special natural or supernatural gifts beyond my talents and natural strengths. | 0.459 | 0 | 0 | 0 | 0.614 |
| SIP004 | Based on your experiences within the past year, please tell me how much you agree or disagree with the following statements: I think that I might be able to predict the future. | 0.417 | 0 | 0 | 0 | 0.576 |
| SIP008 | Based on your experiences within the past year, please tell me how much you agree or disagree with the following statements: I have thought that it might be possible that other people can read my mind, or that I can read others' minds | 0.474 | 0 | 0 | 0 | 0.566 |
| SIP005 | Based on your experiences within the past year, please tell me how much you agree or disagree with the following statements: I may have felt that there could possibly be something interrupting or controlling my thoughts, feelings, or actions. | 0.550 | 0 | 0 | 0 | 0.556 |
| SIP011 | Based on your experiences within the past year, please tell me how much you agree or disagree with the following statements: I think I might feel like my mind is "playing tricks" on me. | 0.622 | 0 | 0 | 0 | 0.551 |
| SIP012 | Based on your experiences within the past year, please tell me how much you agree or disagree with the following statements.: I have had the experience of hearing faint or clear sounds of people or a person mumbling or talking when there is no one near me. | 0.640 | 0 | 0 | 0 | 0.548 |
| SIP007 | Based on your experiences within the past year, please tell me how much you agree or disagree with the following statements: I think I may get confused at times whether something I experience or perceive may be real or may be just part of my imagination or dreams. | 0.606 | 0 | 0 | 0 | 0.546 |
| SIP013 | Please indicate the extent to which you agree with the following statement about yourself: I think that I may hear my own thoughts being said out loud. | 0.575 | 0 | 0 | 0 | 0.533 |
| SIP006 | Based on your experiences within the past year, please tell me how much you agree or disagree with the following statements: I have had the experience of doing something differently because of my superstitions. | 0.544 | 0 | 0 | 0 | 0.532 |
| SIP003 | Based on your experiences within the past year, please tell me how much you agree or disagree with the following statements: I think that I have felt that there are odd or unusual things going on that I can't explain. | 0.605 | 0 | 0 | 0 | 0.482 |
| SIP009 | Based on your experiences within the past year, please tell me how much you agree or disagree with the following statements self: I wonder if people may be planning to hurt me or even may be about to hurt me. | 0.603 | 0 | 0 | 0 | 0.435 |
| SIP014 | Based on your experiences within the past year, please tell me how much you agree or disagree with the following statements: I have been concerned that I might be "going crazy." | 0.685 | 0 | 0 | 0 | 0.429 |
| PSY001 | Have you ever heard voices when no one was there? | 0.633 | 0 | 0 | 0 | 0.385 |
| PSY029 | Have you ever seen visions or seen things which other people could not see? | 0.582 | 0 | 0 | 0 | 0.288 |
| PSY060 | Have you ever had strange feelings in your body like things were crawling on you or someone touching you and nothing or no one was there? | 0.654 | 0 | 0 | 0 | 0.223 |
| PSY070 | Have you ever believed in things that most other people or your parents don't believe in? | 0.579 | 0 | 0 | 0 | 0.167 |
| PSY050 | Have you ever smelled strange odors other people could not smell? | 0.564 | 0 | 0 | 0 | 0.136 |
| PSY071 | Have you ever believed in things and later found out they weren't true, like people being out to get you, or talking about you behind your back, or controlling what you do or think? | 0.643 | 0 | 0 | 0 | 0.114 |
| PTD009 | Have you ever been very upset by seeing a dead PTD009 body or by seeing pictures of the dead body of somebody you knew well? | 0.414 | 0 | 0 | 0 | 0.026 |
| MAN006 | Have you ever had a time when you felt like you could do almost anything? | 0.705 | 0 | 0 | 0 | –0.062 |
| MAN004 | Have there been times when you kept talking a lot, couldn't stop talking, talked faster than usual, had thoughts faster than usual, or had so many ideas in your head that you could hardly keep track of them? | 0.811 | 0 | 0 | 0 | –0.107 |
| MAN003 | Has there ever been a time when you felt like you hardly needed sleep? | 0.778 | 0 | 0 | 0 | –0.116 |
| MAN005 | Have you ever had a time when you felt much more happy or excited than you usually do when there was nothing special going on? | 0.788 | 0 | 0 | 0 | –0.119 |
| MAN001 | Have there been times when you were much more active, excited or energetic than usual, had problems sitting still, or needed to move around a lot? | 0.807 | 0 | 0 | 0 | –0.153 |
| MAN002 | Has there ever been a time when you felt so full of energy that you couldn't stop doing things and didn't get tired? | 0.819 | 0 | 0 | 0 | –0.165 |
| *ADD = attention deficit disorder; AGR = agoraphobia; CDD = conduct disorder; DEP = depression; GAD = generalized anxiety disorder; MAN = mania; OCD = obsessive-compulsive disorder; ODD = opposition-defiant disorder; PAN = panic; PHB = specific phobia; PSY = Psychosis; SCR = screener; SEP = separation anxiety; SIP = PRIME screen or structured interview for prodromal syndromes; SOC = social anxiety; SUI = suicide ideation | | | | | | |

| **Table S2** Correlations between psychopathology factor scores (bifactor model) and cognitive accuracy/reaction time scores | | | | | |
| --- | --- | --- | --- | --- | --- |
|  | General psychopathology | Anxious-Misery | Externalizing | Fear | Psychosis-Spectrum |
| Anxious-Misery | 0.113 |  |  |  |  |
| Externalizing | 0.110 | 0.063 |  |  |  |
| Fear | 0.015 | 0.178 | 0.027 |  |  |
| Psychosis-Spectrum | 0.005 | –0.005 | –0.080 | 0.009 |  |
| Abstraction | –0.078 | –0.011 | –0.074 | –0.044 | 0.013 |
| Attention | –0.075 | 0.026 | –0.072 | –0.065 | –0.044 |
| Working memory | –0.088 | 0.024 | –0.074 | –0.032 | –0.011 |
| Face memory | –0.038 | 0.058 | –0.051 | –0.003 | –0.061 |
| Spatial memory | –0.051 | 0.007 | –0.044 | –0.012 | 0.008 |
| Verbal memory | –0.022 | 0.013 | –0.034 | 0.002 | 0.009 |
| Verbal reasoning | –0.103 | 0.073 | –0.112 | –0.067 | 0.015 |
| Nonverbal reasoning | –0.081 | 0.061 | –0.064 | –0.028 | –0.001 |
| Spatial processing | –0.090 | 0.026 | –0.092 | –0.030 | –0.016 |
| Age differentiation | –0.034 | 0.053 | –0.041 | –0.008 | –0.035 |
| Emotion differentiation | –0.049 | 0.054 | –0.041 | –0.020 | –0.014 |
| Emotion identification | –0.043 | 0.038 | –0.022 | –0.029 | –0.028 |
| WRAT | –0.105 | 0.093 | –0.125 | –0.044 | –0.036 |
| Composite score (*g*) | –0.121 | 0.069 | –0.108 | –0.056 | –0.024 |
| Abstraction | 0.008 | 0.010 | –0.013 | 0.058 | 0.003 |
| Attention | –0.039 | 0.012 | –0.001 | 0.016 | –0.032 |
| Working memory | 0.012 | –0.011 | 0.034 | 0.001 | –0.022 |
| Face memory | –0.025 | –0.024 | –0.047 | 0.059 | 0.029 |
| Spatial memory | –0.042 | 0.013 | –0.065 | 0.082 | 0.040 |
| Verbal memory | 0.008 | 0.008 | –0.009 | 0.090 | 0.031 |
| Verbal reasoning | 0.056 | –0.016 | 0.043 | 0.045 | 0.009 |
| Nonverbal reasoning | –0.010 | 0.066 | –0.015 | 0.007 | 0.007 |
| Spatial processing | –0.004 | 0.014 | –0.065 | 0.041 | 0.005 |
| Age differentiation | 0.004 | 0.025 | –0.055 | 0.071 | 0.028 |
| Emotion differentiation | 0.012 | 0.021 | –0.004 | 0.052 | 0.005 |
| Emotion identification | 0.052 | 0.002 | 0.035 | 0.034 | 0.020 |
| Sensorimotor speed | –0.003 | –0.001 | 0.018 | –0.063 | –0.012 |
| Motor speed | –0.056 | 0.021 | –0.038 | –0.039 | –0.006 |
| Speed composite score (*gs*) | –0.007 | –0.015 | 0.025 | –0.079 | –0.022 |
| WRAT = Wide Range Achievement Test | | | | | |

**Figure S1** Standard error (A) and reliability (B) plots of factors generated from the confirmatory bifactor model of GOASSESS

| **Table S3** Genotype x Age Interaction Model | |
| --- | --- |
|  | The covariance of residual phenotypes (i.e. after regressing out the effect of covariates) between two individuals *i* and *j* can be decomposed as: |
| (1) | cov*_i,j_* = *k_i,j_ σ^2^_g_* + *I_i,j_ σ^2^_e_* |
|  | where *k_i,j_* is the proportion of alleles that are identical by descent between individuals *i* and *j*, *σ^2^_g_* is the additive genetic component of the total phenotypic variance, *I* is an identity matrix composed of 1s down the diagonal (i.e. for the individual with themselves) and 0s everywhere else, and *σ^2^_e_* is the environmental component of the total phenotypic variance. |
|  | The linear decomposition of the phenotypic covariance can be modified to test for a G×A interaction by modeling the variance components as a function of age: |
| (2) | *σ^2^_g_* = *exp(α_g_ + γ_g_(age_i_ −* $\bar{\text{age}}$*)) exp(α_g_ + γ_g_(age_j_ −* $\bar{\text{age}}$*))* |
|  | and |
| (3) | *σ^2^_e_* = *exp(α_e_ + γ_e_(age_i_ −* $\bar{\text{age}}$*)) exp(α_e_ + γ_e_(age_j_ −* $\bar{\text{age}}$*))* |
|  | where *σ^2^_g_* and *σ^2^_e_* are exponential functions of each individual’s deviation in age from the sample mean $(\bar{\text{age}}$*)*. If *γ_g_* is significantly different from 0, there is evidence for change in genetic variance with age. |
|  | In addition to modeling differences in genetic variance across age, we also allow for the possibility of imperfect correlation in genetic variance across age with a genetic correlation (*ρ_G_*) that is a function of the difference in ages between individuals *i* and *j*: |
| (4) | *ρ_G_ = exp*(−*λ_g_*\|*age_i_* − *age_j_*\|) |
|  | The genetic correlation is 1 if *age_i_* − *age_j_* = 0 or if *λ_g_* = 0. If *λ_g_* is significantly greater than 0, there is evidence for change in genetic correlation with age, suggesting that the relative contributions of each genetic factor change with age. It is possible for *γ_g_* to equal 0, but *λ_g_* to be greater than 0, suggesting that although the magnitude of overall aggregate genetic effects is similar at different ages, the sources of these effects differ. |
|  | Combining the above, the covariance equation for individuals *i* and *j* becomes: |
| (5) | cov*_i,j_* = *k_i,j_ exp(α_g_ + γ_g_(age_i_ −* $\bar{\text{age}}$*)) exp(α_g_ + γ_g_(age_j_ −* $\bar{\text{age}}$*)) exp*(−*λ_g_*\|*age_i_* − *age_j_*\|) + *I_i,j_ exp(α_e_ + γ_e_(age_i_ −* $\bar{\text{age}}$*)) exp(α_e_ + γ_e_(age_j_ −* $\bar{\text{age}}$*)).* |

| **Table S4** Factor loadings for the confirmatory hierarchical model of 112 items from the GOASSESS structured interview | | | | | |
| --- | --- | --- | --- | --- | --- |
| Item* | Question | Anxious-Misery | Externalizing | Fear | Psychosis- Spectrum |
| OCD001 | Have you ever been bothered by thoughts that don't make sense to you, that come over and over again and won't go away, such as concern with harming others or yourself? | 0.835 | 0 | 0 | 0 |
| OCD005 | Have you ever been bothered by thoughts that don't make sense to you, that come over and over again and won't go away, such as feeling that bad things that happened were your fault? | 0.815 | 0 | 0 | 0 |
| OCD006 | Have you ever been bothered by thoughts that don't make sense to you, that come over and over again and won't go away, such as forbidden/bad thoughts? | 0.809 | 0 | 0 | 0 |
| OCD004 | Have you ever been bothered by thoughts that don't make sense to you, that come over and over again and won't go away, such as fear that you would do something/say something bad without intending to? | 0.808 | 0 | 0 | 0 |
| DEP004 | Has there ever been a time when you felt grouchy, irritable, or in a bad mood most of the time even little things would make you mad? | 0.785 | 0 | 0 | 0 |
| DEP001 | Has there ever been a time when you felt sad or depressed most of the time? | 0.769 | 0 | 0 | 0 |
| DEP006 | Has there ever been a time when nothing was fun for you and you just weren't interested in anything? | 0.762 | 0 | 0 | 0 |
| OCD002 | Have you ever been bothered by thoughts that don't make sense to you, that come over and over again and won't go away, such as pictures of violent things? | 0.762 | 0 | 0 | 0 |
| MAN007 | Has there ever been a time when you felt unusually grouchy, cranky, or irritable; when the smallest things would make you really mad? | 0.742 | 0 | 0 | 0 |
| DEP002 | Has there ever been a time when you cried a lot, or felt like crying? | 0.740 | 0 | 0 | 0 |
| PAN004 | Has there ever been a time when all of a sudden, you felt that you were losing control, something terrible was going to happen, that you were going crazy, or going to die? | 0.733 | 0 | 0 | 0 |
| OCD008 | Have you ever been bothered by thoughts that don't make sense to you, that come over and over again and won't go away, such as religious thoughts? | 0.719 | 0 | 0 | 0 |
| SIP032 | Do you ever feel a loss of sense of self or feel disconnected from yourself or your life? | 0.707 | 0 | 0 | 0 |
| SUI002 | Have you ever thought about killing yourself? | 0.707 | 0 | 0 | 0 |
| OCD016 | Have you ever had to do something over and over again - that would have made you feel really nervous if you couldn't do it, like ordering or arranging things? | 0.698 | 0 | 0 | 0 |
| OCD011 | Have you ever had to do something over and over again - that would have made you feel really nervous if you couldn't do it, like cleaning or washing (for example, your hands, house)? | 0.688 | 0 | 0 | 0 |
| OCD014 | Have you ever had to do something over and over again - that would have made you feel really nervous if you couldn't do it, like getting dressed over and over again? | 0.688 | 0 | 0 | 0 |
| OCD003 | Have you ever been bothered by thoughts that don't make sense to you, that come over and over again and won't go away, such as thoughts about contamination/germs/illness? | 0.687 | 0 | 0 | 0 |
| OCD017 | Have you ever had to do something over and over again - that would have made you feel really nervous if you couldn't do it, like doing things over and over again at bedtime, like arranging the pillows, sheets, or other things? | 0.675 | 0 | 0 | 0 |
| OCD013 | Have you ever had to do something over and over again - that would have made you feel really nervous if you couldn't do it, like checking (for example, doors, locks, ovens)? | 0.673 | 0 | 0 | 0 |
| OCD012 | Have you ever had to do something over and over again - that would have made you feel really nervous if you couldn't do it, like counting? | 0.671 | 0 | 0 | 0 |
| PAN003 | Has there ever been a time when all of a sudden you felt very, very scared or uncomfortable - and your chest hurt, you couldn't catch your breath, your heart beat very fast, you felt very shaky, and sweaty/tingly/numb in your hands or feet? | 0.668 | 0 | 0 | 0 |
| OCD015 | Have you ever had to do something over and over again - that would have made you feel really nervous if you couldn't do it, like going in and out a door over and over again? | 0.651 | 0 | 0 | 0 |
| OCD007 | Have you ever been bothered by thoughts that don't make sense to you, that come over and over again and won't go away, such as need for symmetry/exactness? | 0.638 | 0 | 0 | 0 |
| SUI001 | Have you ever thought a lot about death or dying? | 0.628 | 0 | 0 | 0 |
| PAN001 | Have you ever had a panic attack? | 0.622 | 0 | 0 | 0 |
| GAD002 | Do you tend to worry a lot more than most children/people your age? | 0.597 | 0 | 0 | 0 |
| SIP033 | Has anyone pointed out to you that you are less emotional or connected to people than you used to be? | 0.596 | 0 | 0 | 0 |
| GAD001 | Have you ever been a worrier? | 0.558 | 0 | 0 | 0 |
| SCR007 | Have you ever had to go to a hospital and stay overnight because of problems with your mood, feelings, or how you were acting? | 0.557 | 0 | 0 | 0 |
| OCD019 | Do you feel the need to do things just right (like they have to be perfect)? | 0.550 | 0 | 0 | 0 |
| OCD018 | Have you ever saved up so many things that people complained or they got in the way? | 0.542 | 0 | 0 | 0 |
| SEP510 | Did you ever worry/have bad dreams about something terrible happening to you or your mother/father/guardian so that you would not see them again? | 0.527 | 0 | 0 | 0 |
| SCR001 | Have you ever talked to a counselor, psychologist, social worker, psychiatrist or some other professional about your feelings or problems with your mood or behaviors? | 0.514 | 0 | 0 | 0 |
| SIP039 | Within the past 6 months, are you having a harder time getting normal activities done? | 0.447 | 0 | 0 | 0 |
| ADD011 | Have you often had trouble paying attention or keeping your mind on your school, work, chores, or other activities that you were doing? | 0 | 0.822 | 0 | 0 |
| ADD016 | Have you often had people tell you that you did not seem to be listening when they spoke to you or that you were daydreaming? | 0 | 0.821 | 0 | 0 |
| ADD012 | Have you often had problems following instructions and often fail to finish school, work, or other things you meant to get done? | 0 | 0.811 | 0 | 0 |
| ODD002 | Was there a time when you often got into trouble with adults for refusing to do what they told you to do or for breaking rules at home/school/work? | 0 | 0.796 | 0 | 0 |
| ODD003 | Did you often annoy other people on purpose or blame other people for your mistakes (excluding siblings)? | 0 | 0.776 | 0 | 0 |
| ODD006 | Were you often irritable or grouchy, or did you often get angry because you thought that things were unfair? | 0 | 0.759 | 0 | 0 |
| ODD001 | Was there a time when you often did things that got you into trouble with adults such as losing your temper, arguing with or talking back to adults, or being grouchy or irritable with them? | 0 | 0.751 | 0 | 0 |
| ADD013 | Have you often disliked, avoided, or put off school or homework (or any other activity requiring concentration) | 0 | 0.748 | 0 | 0 |
| CDD005 | Did you often bully others (hitting, threatening or scaring someone who was younger or smaller), threaten or frighten someone on purpose, or often start physical fights with others? | 0 | 0.740 | 0 | 0 |
| ADD022 | Have you often joined other people's conversations or had trouble waiting your turn (e.g., waiting in line, waiting for a teacher to call on you in class)? | 0 | 0.738 | 0 | 0 |
| ADD021 | Have you often blurted out answers to other people's questions before they finished speaking or interrupted people abruptly? | 0 | 0.730 | 0 | 0 |
| ADD014 | Have you often lost things you needed for school or projects at home (assignments or books) or made careless mistakes in school work or other activities? | 0 | 0.726 | 0 | 0 |
| ODD005 | Did you ever get into trouble for getting even with other people by doing things to hurt them, telling lies about them, or messing up their things? | 0 | 0.725 | 0 | 0 |
| ADD015 | Have you often had trouble making plans, doing things that had to be done in a certain kind of order or that had a lot of different steps? | 0 | 0.722 | 0 | 0 |
| ADD020 | Have you often had difficulty sitting still for more than a few minutes at a time, even after being asked to stay seated, or have you often fidgeted with your hands or feet or wiggled in your seat? | 0 | 0.719 | 0 | 0 |
| CDD003 | Did you ever set fires, break into cars, or destroy someone else's property on purpose? | 0 | 0.705 | 0 | 0 |
| CDD008 | Have you ever threatened someone? | 0 | 0.690 | 0 | 0 |
| CDD001 | Was there ever a time when you often did things that got you into trouble with adults like lying or stealing (something worth more than $5) from family, others, or stores? | 0 | 0.670 | 0 | 0 |
| CDD007 | Have you ever tried to hurt someone with a weapon (a bat, brick, broken bottle, knife, or gun)? | 0 | 0.661 | 0 | 0 |
| CDD006 | Have you ever been physically cruel to an animal or person (on purpose)? | 0 | 0.651 | 0 | 0 |
| SCR008 | Have you or anyone else (like your friends, parents, or teachers) ever thought you needed help because of problems with your mood, feelings, or how you were acting? | 0 | 0.573 | 0 | 0 |
| CDD002 | Have you ever skipped school, stayed out at night later than you were supposed to (more than 2 hours), or run away from home overnight? | 0 | 0.531 | 0 | 0 |
| SCR006 | Are you currently taking medication because of your emotions and/or behaviors? | 0 | 0.525 | 0 | 0 |
| CDD004 | Do you have a probation officer or have you ever been on probation? | 0 | 0.462 | 0 | 0 |
| SIP038 | Within the past 6 months, are you having a harder time getting your work or schoolwork done? | 0 | 0.418 | 0 | 0 |
| AGR006 | Have you ever been very nervous or afraid of traveling away from home? | 0 | 0 | 0.774 | 0 |
| AGR002 | Have you ever been very nervous or afraid of going to public places (such as a store or shopping mall)? | 0 | 0 | 0.767 | 0 |
| AGR008 | Have you ever been very nervous or afraid of using public transportation like a bus, train, or subway? | 0 | 0 | 0.763 | 0 |
| SOC005 | Was there ever a time in your life when you felt afraid or uncomfortable because you were the center of attention and were concerned something embarrassing might happen and you felt very afraid or felt uncomfortable? | 0 | 0 | 0.762 | 0 |
| AGR001 | Have you ever been very nervous or afraid of being in crowds (for example, a classroom, cafeteria, restaurant, or movie theater)? | 0 | 0 | 0.755 | 0 |
| SOC004 | Was there ever a time in your life when you felt afraid or uncomfortable acting, performing, giving a talk/speech, playing a sport or doing a musical performance, or taking an important test or exam (even though you studied enough)? | 0 | 0 | 0.750 | 0 |
| AGR003 | Have you ever been very nervous or afraid of being in an open field? | 0 | 0 | 0.725 | 0 |
| AGR005 | Have you ever been very nervous or afraid of traveling by yourself? | 0 | 0 | 0.722 | 0 |
| SOC001 | Was there ever a time in your life when you felt afraid or uncomfortable or really, really shy with people, like meeting new people, going to parties, or eating or drinking, writing or doing homework in front of others? | 0 | 0 | 0.721 | 0 |
| SOC003 | Was there ever a time in your life when you felt afraid or uncomfortable when you had to do something in front of a group of people, like speaking in class? | 0 | 0 | 0.703 | 0 |
| AGR004 | Have you ever been very nervous or afraid of going over bridges or through tunnels? | 0 | 0 | 0.688 | 0 |
| SOC002 | Was there ever a time in your life when you felt afraid or uncomfortable talking on the telephone or with people your own age who you don't know very well? | 0 | 0 | 0.669 | 0 |
| AGR007 | Have you ever been very nervous or afraid of traveling in a car? | 0 | 0 | 0.574 | 0 |
| PHB007 | Have you ever been very nervous or afraid of flying or airplanes? | 0 | 0 | 0.572 | 0 |
| PHB006 | Have you ever been very nervous or afraid of closed spaces, like elevators or closets? | 0 | 0 | 0.540 | 0 |
| SEP509 | When you knew that you were going to be away from home or mother/father/guardian, have you ever become very upset and worried (e.g., when you learned your mother/father/guardian were going on an upcoming trip or night out)? | 0 | 0 | 0.512 | 0 |
| PHB002 | Have you ever been very nervous or afraid of being in really high places, like a roof or tall building? | 0 | 0 | 0.500 | 0 |
| SEP500 | Since you were 5 years old, has there ever been a time when you had a lot of worries about your attachment figures (mother, father, guardian) and were very upset or got sick (for example, felt sick to your stomach, headaches, thrown-up) when you were away from them? | 0 | 0 | 0.481 | 0 |
| PHB003 | Have you ever been very nervous or afraid of water or situations involving water, such as a swimming pool, lake, or ocean? | 0 | 0 | 0.480 | 0 |
| SEP508 | Has there ever been a time when you wanted to stay home from school or not go to other places without your mother, father, or guardian? | 0 | 0 | 0.475 | 0 |
| PHB004 | Have you ever been very nervous or afraid of storms, thunder, or lightning? | 0 | 0 | 0.460 | 0 |
| PHB001 | Have you ever been very nervous or afraid of animals or bugs, like dogs, snakes, or spiders? | 0 | 0 | 0.449 | 0 |
| PHB008 | Have you ever been very nervous or afraid of specific things or situations? | 0 | 0 | 0.437 | 0 |
| SEP511 | Have you ever been scared to be alone in your room (or any place in your house) or did you need your mother/father/guardian to stay with you while you fell asleep? | 0 | 0 | 0.345 | 0 |
| PHB005 | Have you ever been very nervous or afraid of doctors, needles, or blood? | 0 | 0 | 0.339 | 0 |
| SIP012 | Based on your experiences within the past year, please tell me how much you agree or disagree with the following statements.: I have had the experience of hearing faint or clear sounds of people or a person mumbling or talking when there is no one near me. | 0 | 0 | 0 | 0.838 |
| SIP014 | Based on your experiences within the past year, please tell me how much you agree or disagree with the following statements: I have been concerned that I might be "going crazy." | 0 | 0 | 0 | 0.813 |
| SIP011 | Based on your experiences within the past year, please tell me how much you agree or disagree with the following statements: I think I might feel like my mind is "playing tricks" on me. | 0 | 0 | 0 | 0.811 |
| SIP007 | Based on your experiences within the past year, please tell me how much you agree or disagree with the following statements: I think I may get confused at times whether something I experience or perceive may be real or may be just part of my imagination or dreams. | 0 | 0 | 0 | 0.786 |
| SIP013 | Please indicate the extent to which you agree with the following statement about yourself: I think that I may hear my own thoughts being said out loud. | 0 | 0 | 0 | 0.783 |
| SIP005 | Based on your experiences within the past year, please tell me how much you agree or disagree with the following statements: I may have felt that there could possibly be something interrupting or controlling my thoughts, feelings, or actions. | 0 | 0 | 0 | 0.765 |
| SIP003 | Based on your experiences within the past year, please tell me how much you agree or disagree with the following statements: I think that I have felt that there are odd or unusual things going on that I can't explain. | 0 | 0 | 0 | 0.753 |
| MAN004 | Have there been times when you kept talking a lot, couldn't stop talking, talked faster than usual, had thoughts faster than usual, or had so many ideas in your head that you could hardly keep track of them? | 0 | 0 | 0 | 0.750 |
| MAN002 | Has there ever been a time when you felt so full of energy that you couldn't stop doing things and didn't get tired? | 0 | 0 | 0 | 0.745 |
| SIP009 | Based on your experiences within the past year, please tell me how much you agree or disagree with the following statements self: I wonder if people may be planning to hurt me or even may be about to hurt me. | 0 | 0 | 0 | 0.741 |
| SIP006 | Based on your experiences within the past year, please tell me how much you agree or disagree with the following statements: I have had the experience of doing something differently because of my superstitions. | 0 | 0 | 0 | 0.740 |
| PSY001 | Have you ever heard voices when no one was there? | 0 | 0 | 0 | 0.738 |
| MAN003 | Has there ever been a time when you felt like you hardly needed sleep? | 0 | 0 | 0 | 0.736 |
| MAN001 | Have there been times when you were much more active, excited or energetic than usual, had problems sitting still, or needed to move around a lot? | 0 | 0 | 0 | 0.733 |
| MAN005 | Have you ever had a time when you felt much more happy or excited than you usually do when there was nothing special going on? | 0 | 0 | 0 | 0.733 |
| PSY060 | Have you ever had strange feelings in your body like things were crawling on you or someone touching you and nothing or no one was there? | 0 | 0 | 0 | 0.714 |
| SIP008 | Based on your experiences within the past year, please tell me how much you agree or disagree with the following statements: I have thought that it might be possible that other people can read my mind, or that I can read others' minds | 0 | 0 | 0 | 0.705 |
| SIP010 | Based on your experiences within the past year, please tell me how much you agree or disagree with the following statements: I believe that I have special natural or supernatural gifts beyond my talents and natural strengths. | 0 | 0 | 0 | 0.704 |
| MAN006 | Have you ever had a time when you felt like you could do almost anything? | 0 | 0 | 0 | 0.691 |
| PSY029 | Have you ever seen visions or seen things which other people could not see? | 0 | 0 | 0 | 0.654 |
| PSY071 | Have you ever believed in things and later found out they weren't true, like people being out to get you, or talking about you behind your back, or controlling what you do or think? | 0 | 0 | 0 | 0.653 |
| SIP004 | Based on your experiences within the past year, please tell me how much you agree or disagree with the following statements: I think that I might be able to predict the future. | 0 | 0 | 0 | 0.649 |
| PSY070 | Have you ever believed in things that most other people or your parents don't believe in? | 0 | 0 | 0 | 0.623 |
| PSY050 | Have you ever smelled strange odors other people could not smell? | 0 | 0 | 0 | 0.593 |
| SIP027 | Do people ever tell you that they can't understand you? | 0 | 0 | 0 | 0.515 |
| SIP028 | Do people ever seem to have difficulty understanding you? | 0 | 0 | 0 | 0.514 |
| PTD009 | Have you ever been very upset by seeing a dead PTD009 body or by seeing pictures of the dead body of somebody you knew well? | 0 | 0 | 0 | 0.389 |
| General Factor | | 0.962 | 0.710 | 0.783 | 0.836 |
| *ADD = attention deficit disorder; AGR = agoraphobia; CDD = conduct disorder; DEP = depression; GAD = generalized anxiety disorder; MAN = mania; OCD = obsessive-compulsive disorder; ODD = opposition-defiant disorder; PAN = panic; PHB = specific phobia; PSY = Psychosis; SCR = screener; SEP = separation anxiety; SIP = PRIME screen or structured interview for prodromal syndromes; SOC = social anxiety; SUI = suicide ideation | | | | | |

| **Table S5** Correlations between psychopathology factor scores (hierarchical model) and cognitive accuracy/reaction time scores | | | | | |
| --- | --- | --- | --- | --- | --- |
|  | General psychopathology | Anxious-Misery | Externalizing | Fear | Psychosis-Spectrum |
| Anxious-Misery | 0.989 |  |  |  |  |
| Externalizing | 0.805 | 0.759 |  |  |  |
| Fear | 0.729 | 0.679 | 0.524 |  |  |
| Psychosis-Spectrum | 0.861 | 0.802 | 0.645 | 0.633 |  |
| Abstraction | –0.077 | –0.094 | –0.086 | –0.069 | –0.060 |
| Attention | –0.074 | –0.096 | –0.098 | –0.058 | –0.085 |
| Working memory | –0.076 | –0.104 | –0.085 | –0.061 | –0.090 |
| Face memory | –0.019 | –0.064 | –0.029 | –0.003 | –0.058 |
| Spatial memory | –0.039 | –0.067 | –0.048 | –0.032 | –0.036 |
| Verbal memory | –0.020 | –0.040 | –0.019 | –0.017 | –0.020 |
| Verbal reasoning | –0.075 | –0.136 | –0.110 | –0.054 | –0.087 |
| Nonverbal reasoning | –0.055 | –0.090 | –0.070 | –0.038 | –0.077 |
| Spatial processing | –0.075 | –0.117 | –0.082 | –0.059 | –0.089 |
| Age differentiation | –0.015 | –0.044 | –0.027 | –0.002 | –0.044 |
| Emotion differentiation | –0.029 | –0.046 | –0.047 | –0.016 | –0.051 |
| Emotion identification | –0.025 | –0.039 | –0.041 | –0.014 | –0.047 |
| WRAT | –0.076 | –0.151 | –0.097 | –0.050 | –0.116 |
| Composite score (*g*) | –0.093 | –0.147 | –0.121 | –0.068 | –0.119 |
| Abstraction | 0.014 | –0.008 | 0.047 | 0.009 | 0.012 |
| Attention | –0.023 | –0.023 | –0.011 | –0.019 | –0.045 |
| Working memory | 0.009 | 0.036 | 0.006 | 0.008 | –0.002 |
| Face memory | –0.017 | –0.041 | 0.022 | –0.022 | 0.000 |
| Spatial memory | –0.020 | –0.067 | 0.028 | –0.021 | –0.020 |
| Verbal memory | 0.026 | 0.007 | 0.073 | 0.019 | 0.021 |
| Verbal reasoning | 0.059 | 0.073 | 0.071 | 0.049 | 0.059 |
| Nonverbal reasoning | 0.017 | –0.016 | 0.000 | 0.025 | –0.002 |
| Spatial processing | 0.005 | –0.046 | 0.021 | 0.007 | 0.004 |
| Age differentiation | 0.017 | –0.033 | 0.050 | 0.016 | 0.011 |
| Emotion differentiation | 0.025 | 0.011 | 0.046 | 0.023 | 0.018 |
| Emotion identification | 0.056 | 0.060 | 0.061 | 0.048 | 0.059 |
| Sensorimotor speed | –0.049 | –0.067 | –0.070 | –0.037 | –0.057 |
| Motor speed | –0.013 | 0.009 | –0.048 | –0.009 | –0.007 |
| Speed composite score (*gs*) | –0.024 | 0.007 | –0.059 | –0.019 | –0.020 |
| WRAT = Wide Range Achievement Test | | | | | |

**Figure S2** Standard error (A) and reliability (B) plots of factors generated from the confirmatory hierarchical model

**Figure S3** Heritability estimates for psychopathology factors generated from hierarchical model of GOASSESS*

*Error bars represent standard errors (SEs)

| **Table S6** Heritability estimates of psychopathology factors (hierarchical model), genetic and phenotypic correlations between heritable factors and cognitive measures | | | | | | | | | | | | | | | | |
| --- | --- | --- | --- | --- | --- | --- | --- | --- | --- | --- | --- | --- | --- | --- | --- | --- |
| Category | Trait |  |  | General psychopathology | | | | Anxious-Misery | | | | | Externalizing | | | |
|  |  | *h^2^* | *p* | *ρ_g_* | *p* | *ρ_p_* | *p* | *ρ_g_* | | *p* | *ρ_p_* | *p* | *ρ_g_* | *p* | *ρ_p_* | *p* |
| Psychopathology | General psychopathology | **0.37** | **0.001** | - | - | - | - | - | | - | - | - | - | - | - | - |
|  | Anxious-Misery | **0.35** | **0.002** | **0.99** | **4×10^–127^** | **0.99** | **1×10^–307^** | - | | - | - | - | - | - | - | - |
|  | Externalizing | **0.58** | **1×10^–7^** | **0.88** | **6×10^–5^** | **0.75** | **1×10^–307^** | **0.82** | | **3×10^–4^** | **0.70** | **1×10^–307^** | - | - | - | - |
|  | Fear | 0.25 | 0.011 | - | - | - | - | - | | - | - | - | - | - | - | - |
|  | Psychosis-Spectrum | 0.12 | 0.161 | - | - | - | - | - | | - | - | - | - | - | - | - |
| Accuracy | Abstraction | - | - | - | - | - | - | - | | - | - | - | - | - | - | - |
|  | Attention | - | - | –0.25 | 0.221 | **–0.075** | **4×10^–7^** | –0.22 | | 0.276 | **–0.06** | **6×10^–5^** | –0.14 | 0.352 | **–0.10** | **6×10^–11^** |
|  | Working memory | - | - | –0.14 | 0.592 | **–0.072** | **1×10^–6^** | –0.05 | | 0.835 | **–0.06** | **9×10^–5^** | –0.40 | 0.039 | **–0.10** | **5×10^–11^** |
|  | Face memory | - | - | –0.06 | 0.746 | –0.019 | 0.200 | 0.00 | | 0.992 | 0.00 | 0.879 | –0.32 | 0.012 | **–0.06** | **2×10^–5^** |
|  | Spatial memory | - | - | –0.23 | 0.280 | **–0.040** | **0.006** | –0.21 | | 0.324 | –0.03 | 0.025 | –0.18 | 0.248 | **–0.07** | **4×10^–6^** |
|  | Verbal memory | - | - | –0.04 | 0.855 | –0.019 | 0.191 | 0.04 | | 0.850 | –0.02 | 0.284 | –0.10 | 0.514 | **–0.04** | **0.014** |
|  | Verbal reasoning | - | - | –0.10 | 0.601 | **–0.076** | **2×10^–7^** | –0.04 | | 0.830 | **–0.06** | **2×10^–4^** | **–0.43** | **0.001** | **–0.13** | **3×10^–20^** |
|  | Nonverbal reasoning | - | - | 0.01 | 0.938 | **–0.054** | **2×10^–4^** | 0.04 | | 0.814 | **–0.04** | **0.009** | –0.22 | 0.098 | **–0.09** | **9×10^–10^** |
|  | Spatial reasoning | - | - | –0.05 | 0.783 | **–0.075** | **3×10^–7^** | 0.05 | | 0.819 | **–0.06** | **6×10^–5^** | –0.37 | 0.013 | **–0.12** | **2×10^–15^** |
|  | Age differentiation | - | - | - | - | - | - | - | | - | - | - | - | - | - | - |
|  | Emotion differentiation | - | - | –0.23 | 0.424 | –0.030 | 0.040 | –0.22 | | 0.464 | –0.02 | 0.237 | –0.26 | 0.227 | **–0.05** | **0.001** |
|  | Emotion identification | - | - | 0.30 | 0.202 | –0.026 | 0.081 | 0.33 | | 0.162 | –0.01 | 0.326 | 0.11 | 0.530 | **–0.04** | **0.008** |
|  | WRAT | - | - | –0.01 | 0.926 | **–0.078** | **9×10^–8^** | 0.04 | | 0.759 | **–0.05** | **0.001** | **–0.25** | **0.005** | **–0.15** | **2×10^–25^** |
|  | Composite score (*g*) | - | - | –0.14 | 0.293 | **–0.094** | **2×10^–10^** | –0.07 | | 0.594 | **–0.07** | **3×10^–6^** | **–0.37** | **1×10^–4^** | **–0.15** | **4×10^–24^** |
| Reaction time | Abstraction | - | - | - | - | - | - | - | | - | - | - | - | - | - | - |
|  | Attention | - | - | –0.26 | 0.237 | –0.019 | 0.190 | –0.25 | | 0.274 | –0.02 | 0.303 | –0.12 | 0.472 | –0.02 | 0.233 |
|  | Working memory | - | - | - | - | - | - | - | | - | - | - | - | - | - | - |
|  | Face memory | - | - | –0.08 | 0.736 | –0.017 | 0.260 | –0.10 | | 0.698 | –0.02 | 0.141 | –0.04 | 0.846 | **–0.04** | **0.004** |
|  | Spatial memory | - | - | –0.01 | 0.967 | –0.020 | 0.175 | –0.06 | | 0.832 | –0.02 | 0.144 | 0.10 | 0.599 | **–0.07** | **4×10^–6^** |
|  | Verbal memory | - | - | 0.26 | 0.246 | 0.026 | 0.076 | 0.18 | | 0.428 | 0.02 | 0.187 | 0.31 | 0.073 | 0.01 | 0.697 |
|  | Verbal reasoning | - | - | - | - | - | - | - | | - | - | - | - | - | - | - |
|  | Nonverbal reasoning | - | - | - | - | - | - | - | | - | - | - | - | - | - | - |
|  | Spatial reasoning | - | - | –0.09 | 0.743 | 0.005 | 0.721 | –0.07 | | 0.819 | 0.01 | 0.642 | –0.18 | 0.379 | **–0.05** | **0.001** |
|  | Age differentiation | - | - | 0.14 | 0.580 | 0.017 | 0.237 | 0.13 | | 0.614 | 0.02 | 0.267 | 0.22 | 0.228 | **–0.03** | **0.023** |
|  | Emotion differentiation | - | - | 0.03 | 0.914 | 0.026 | 0.081 | –0.02 | | 0.937 | 0.02 | 0.121 | 0.18 | 0.317 | 0.01 | 0.479 |
|  | Emotion identification | - | - | 0.27 | 0.297 | **0.056** | **1×10^–4^** | 0.22 | | 0.383 | **0.05** | **0.001** | 0.42 | 0.033 | **0.06** | **4×10^–5^** |
|  | Sensorimotor speed | - | - | 0.17 | 0.391 | –0.013 | 0.393 | 0.22 | | 0.273 | –0.01 | 0.562 | 0.04 | 0.783 | 0.01 | 0.483 |
|  | Motor speed | - | - | 0.03 | 0.873 | **–0.050** | **0.001** | 0.07 | | 0.732 | –0.04 | 0.009 | –0.34 | 0.032 | **–0.07** | **2×10^–6^** |
|  | Speed composite score (*gs*) | - | - | –0.08 | 0.710 | –0.024 | 0.096 | –0.04 | | 0.836 | –0.02 | 0.176 | –0.17 | 0.322 | 0.01 | 0.628 |
| Bolded estimates significant after correction for multiple testing (FDR = 0.05)  WRAT = Wide Range Achievement Test | | | | | | | | |  |  |  |  |  |  |  |  |

**Figure S4** Estimated genetic variance, environmental variance and heritability by age
